# Considerations on gradual glutamate accumulation related to cognitive task performance

**DOI:** 10.1177/0271678X221139550

**Published:** 2022-11-11

**Authors:** Harald E Möller

**Affiliations:** Max Planck Institute for Human Cognitive and Brain Sciences, Leipzig, Germany

**Keywords:** Cognitive fatigue, excitotoxicity, glutamate, magnetic resonance spectroscopy, neurotransmitter cycling

## Abstract

Long-lasting activities with high demand in cognitive control are known to result in cognitive fatigue. However, the reason for control cost inflation remains elusive. A neurometabolic account was proposed in a recent study combining magnetic resonance spectroscopy (MRS) with daylong execution of behavioral tasks. It suggests that control cost during high-demand work is related to the necessity of recycling potentially toxic substances, specifically glutamate, which may accumulate extracellularly. As MRS provides estimates of metabolite concentrations, further evaluations are possible how well this hypothesis fits with fundamental consequences from the dynamic equilibrium of intercompartmental glutamate distributions.

In experiments involving multiple sessions of proton MRS in subjects performing either high- or low-demand cognitive tasks, Wiehler et al.^1^ observed higher levels of cerebral glutamate in the lateral prefrontal cortex in the ‘high-demand’ compared to the ‘low-demand’ cohort, whereas there was no group difference in a visual reference region. Diffusion-weighted MRS indicated a higher mobility of glutamate plus glutamine (quantified together), which was interpreted *qualitatively* as glutamate accumulation in the extracellular space, where diffusion of small metabolites is typically faster than intracellularly. As MRS can measure (on a centimeter scale) metabolite concentrations, it is possible to evaluate this hypothesis *quantitatively*, while considering that such observations lack specificity to only one compartment, metabolic pathway or kinetic pool. Figure 4 in Wiehler et al.^1^ reports a difference of approximately 10–11% in the metabolite ratio of glutamate and total creatine (taken as internal concentration reference) between the high- and low-demand groups during the final 75 min of a 6.25-h experiment. This difference is proposed to reflect gradual glutamate accumulation associated with greater release in the high-demand task combined with insufficient clearance during daylong execution.

A 10% increase of the glutamate level is in the upper range of changes observed by functional MRS during sustained stimulation, which normalized within minutes.^2–4^ Assuming a typical baseline concentration of [Glu] ≈ 10 µmol/g wet tissue detected by MRS^2^ and ignoring that only a small portion of the spectroscopic volume corresponds to activated cortex, this corresponds to a change by ≈1 mM. Besides neurochemical consequences of activation, metabolic compartmentation and MRS ‘visibility’ must be considered in interpretations of task-related MRS responses. Briefly, cerebral glutamate is predominantly distributed inside the cytoplasm, with a larger concentration (10–15 mM) in neurons compared to astrocytes (≈1 mM).^3–6^ Glutamate sequestered in synaptic vesicles is present at high concentrations (≈100 mM),^6^ however, this fraction does not seem to contribute to the MRS signal due to restricted mobility in a tightly packed microenvironment.^7^ Comparted to the intracellular compartments, baseline concentrations in the extracellular space, which represents approximately 20% of the cortical volume, are very low (≈3 µM).^6^ Considering this compartmentalization, a rough estimation yields an increase in [Glu] by only 0.15%, even in the extreme case of the simultaneous exocytotic release of presumably ‘invisible’ vesicular Glu into the ‘visible’ (peri-)synaptic extracellular space in *all* synapses.^4^ Such alterations are far below current MRS detection limits. Moreover, glutamate redistribution equivalent to 1 mM total cortical glutamate and extracellular accumulation (on a timescale of hours) would correspond to [Glu] ≈ 5 mM in the extracellular compartment. This is two orders of magnitude above neurotoxic levels (tens of µM), which lead to cell death if persisting for a few minutes.^5^

While these simplified considerations cannot capture all aspects of glutamate distribution and metabolic fluxes, the estimated orders of magnitude should be realistic. This leads to the following conclusions: (*i*) Extracellular [Glu] at baseline or under sustained neuronal activation is orders of magnitude too low to be brought in line with the concentration difference observed by Wiehler et al.^1^ (*ii*) Current MRS techniques are not sensitive enough to probe physiological [Glu] changes in the *extracellular* space. (*iii*) Given the minimal extracellular contribution to [Glu] detected by MRS, glutamate diffusivity changes may not be attributed to the extracellular compartment. These considerations do not rule out extracellular [Glu] alterations *below* excitotoxic levels. However, this cannot be deduced from MRS results beyond speculation. Alternatively, the diffusion experiments might also reflect glutamine changes considering the difficulty to separate glutamate from glutamine at 3 T. Unlike glutamate, glutamine is nontoxic and could accumulate extracellularly with minimal physiological consequences.

Convincing evidence relates increased [Glu] during sustained activation to an increased tricarboxylic acid cycle (TCA) rate to which glutamate is linked via dynamic exchange with α-ketoglutarate.^3^ Given opposite concentration changes of glutamate and aspartate under such conditions, this highlights glutamate’s ‘metabolic’ rather than its ‘transmitter role’. Glutamate accumulation may result from inhibition of glutamine synthetase, which is, however, believed to augment glutamate oxidation, or by *de novo* synthesis in astrocytes.^3,8^ Anaplerotic pyruvate carboxylase activity adds net carbon to the astrocytic TCA cycle contributing to extracellular glutamine release for use by neurons.^8^ Another relevant (glial) glutamine pool is probably invisible to proton MRS,^9^ which might be metabolized for energy.^8^ Taken together, adaptations in anaplerosis and pyruvate recycling in astrocytes could explain glutamate/glutamine changes seen with MRS. Glutamate accumulation induced by feeding or glucose infusion was recently reported.^10^ Therefore, altered glucose availability in the circulation caused by a stress-related hormonal response during task execution could also contribute to increased brain [Glu].

Wiehler et al. did not integrate baseline measurements preceding task execution but acquired MRS data only during stimulation. A task-related [Glu] *increase* was only indirectly obtained from the group difference, which was driven by a [Glu] *decrease* in the low-demand cohort. An alternative interpretation might be an adaptation of the neuronal network with gradually reduced synaptic transmission and, hence, declining demand for elevating oxidative metabolism. Such habituation evolving into an automatic routine during daylong performance may occur during the low-demand task but remain inefficient for the high-demand condition.

In summary, the crucial requirement for an efficient and rapid glutamate removal from the extracellular space and long-term maintenance of low, non-toxic extracellular concentrations suggests that potentially sustained extracellular glutamate accumulation must be orders of magnitude below alterations observed by MRS. Therefore, the authors’ alternative hypothesis that inflated cost of cognitive control may exhaust some metabolic resource remains as a plausible scenario.

## References

[1] Wiehler A Branzoli F Adanyeguh I , et al. A neuro-metabolic account of why daylong cognitive work alters the control of economic decisions. Curr Biol 2022; 32: 3564–3575.e5. 35961314 10.1016/j.cub.2022.07.010

[2] Mangia S Tkác I Gruetter R , et al. Sustained neuronal activation raises oxidative metabolism to a new steady-state level: evidence from ^1^H NMR spectroscopy in the human visual cortex. J Cereb Blood Flow Metab 2007; 27: 1055–1063. 17033694 10.1038/sj.jcbfm.9600401

[3] Mangia S Giove F DiNuzzo M. Metabolic pathways and activity-dependent modulation of glutamate concentration in the human brain. Neurochem Res 2012; 37: 2554–2561. 22846967 10.1007/s11064-012-0848-4 PMC3489977

[4] Martínez-Maestro M Labadie C Möller HE. Dynamic metabolic changes in human visual cortex in regions with positive and negative blood oxygenation level-dependent response. J Cereb Blood Flow Metab 2019; 39: 2295–2307. 30117749 10.1177/0271678X18795426 PMC6827122

[5] Attwell D Barbour B Szatkowski M. Neurovesicular release of neurotransmitter. Neuron 1993; 11: 401–407. 8104430 10.1016/0896-6273(93)90145-h

[6] Danbolt NC. Glutamate uptake. Prog Neurobiol 2001; 65: 1–105. 11369436 10.1016/s0301-0082(00)00067-8

[7] Kauppinen RA Pirttilä TRM Auriola SOK , et al. Compartmentation of cerebral glutamate in situ as detected by ^1^H/^13^C n.m.r. Biochem J 1994; 298: 121–127. 7907470 10.1042/bj2980121 PMC1137991

[8] McKenna MC. The glutamate-glutamine cycle is not stoichiometric: fates of glutamate in brain. J Neurosci Res 2007; 85: 3347–3358. 17847118 10.1002/jnr.21444

[9] Duarte JMN Gruetter R. Glutamatergic and GABAergic energy metabolism measured in the rat brain by ^13^C NMR spectroscopy at 14.1 T. J Neurochem 2013; 126: 579–590. 23745684 10.1111/jnc.12333

[10] Kubota M Kimura Y Shimojo M , et al. Dynamic alterations in the central glutamatergic status following food and glucose intake: in vivo multimodal assessments in humans and animal models. J Cereb Blood Flow Metab 2021; 41: 2928–2943. 34039039 10.1177/0271678X211004150 PMC8545038

